# Supplementary material for: Dynalign II: common secondary structure prediction for RNA homologs with domain insertions
Source: Nucleic Acids Res. 2014 Nov 21;42(22):13939–48. doi: 10.1093/nar/gku1172 (PMC4267632; doi:10.1093/nar/gku1172)
Supplement: SUPPLEMENTARY DATA [file supp_gku1172_nar-02021-z-2014-File012.zip › manual/RNA_class/html/class_two_r_n_a.html]

RNAstructure Classes: TwoRNA Class Reference

- Main Page
- Classes
- Files

- Class List
- Class Hierarchy
- Class Members

# TwoRNA Class Reference

TwoRNA Class.
More...

`#include <TwoRNA.h>`

Inherited by Dynalign\_object.

List of all members.

|  |  |
| --- | --- |
|  |  |
| Public Member Functions | |
|  | TwoRNA (const char sequence1[], const char sequence2[], bool IsRNA=true) |
|  | Constructor - user provides a sequences as c strings. |
|  | TwoRNA (const char filename1[], const int type1, const char filename2[], const int type2, bool IsRNA=true) |
|  | Constructor - user provides a filenames for existing files as a c string. |
|  | TwoRNA () |
| int | SetTemperature (double temperature) |
|  | Set the temperature at which the calculation will be performed in K. |
| double | GetTemperature () |
|  | Get the current folding temperature in K. |
| int | GetErrorCode () |
|  | Return an error code, where a return of zero is no error. |
| char \* | GetErrorMessage (const int error) |
|  | Return error messages based on code from GetErrorCode and other error codes. |
| std::string | GetErrorMessageString (const int error) |
|  | Return error messages based on code from GetErrorCode and other error codes. |
| RNA \* | GetRNA1 () |
|  | return A pointer to the underlying structure class for sequence 1. |
| RNA \* | GetRNA2 () |
|  | return A pointer to the underlying structure class for sequence 2. |
|  | ~TwoRNA () |
|  | Destructor. |
| Public Attributes | |
| char | compoundmessage [COMPOUNDMESSAGELENGTH] |
|  | Provide facility for storing and accessing error messages. |
| Protected Attributes | |
| int | ErrorCodeTwo |
| Private Attributes | |
| RNA \* | rna1 |
| RNA \* | rna2 |

---

## Detailed Description

TwoRNA Class.

The TwoRNA class provides an entry point for all the two sequence prediction routines of RNAstructure. This contains two instances of the RNA class to provide the functionality of RNA. 

---

## Constructor & Destructor Documentation

|  |  |  |  |  |
| --- | --- | --- | --- | --- |
| TwoRNA::TwoRNA | ( | const char | *sequence1*[], |  |
|  |  | const char | *sequence2*[], |  |
|  |  | bool | *IsRNA* = `true` |  |
|  | ) |  |  |  |

Constructor - user provides a sequences as c strings.

Input sequences should contain A,C,G,T,U,a,c,g,t,u,x,X. Capitalization makes no difference. T=t=u=U. If IsRNA is true, the backbone is RNA, so U is assumed. If IsRNA is false, the backbone is DNA, so T is assumed. x=X= nucleotide that neither stacks nor pairs. For now, any unknown nuc is considered 'X'. Both sequences are passed to underlying RNA classes for each sequence.

**Parameters:**
:   |  |  |  |
    | --- | --- | --- |
    |  | *sequence1* | is a NULL terminated c string for sequence 1. |
    |  | *sequence2* | is a NULL terminated c string for sequence 2. |
    |  | *IsRNA* | is a bool that indicates whether these sequences are RNA or DNA. true=RNA. false=DNA. Default is true. Both sequences must have the same backbone. |

|  |  |  |  |  |
| --- | --- | --- | --- | --- |
| TwoRNA::TwoRNA | ( | const char | *filename1*[], |  |
|  |  | const int | *type1*, |  |
|  |  | const char | *filename2*[], |  |
|  |  | const int | *type2*, |  |
|  |  | bool | *IsRNA* = `true` |  |
|  | ) |  |  |  |

Constructor - user provides a filenames for existing files as a c string.

The existing files, specified by filenames, can either be a ct file, a sequence, or an RNAstructure save file. Therefore, the user provides a flag for the file type: type = 1 => .ct file, type = 2 => .seq file, type = 3 => partition function save (.pfs) file, type = 4 => folding save file (.sav). The file opening is performed by the constructors for the RNA classes that underlie each sequence. This constructor generates internal error codes that can be accessed by GetErrorCode() after the constructor is called. 0 = no error. The errorcode can be resolved to a c string using GetErrorMessage. Note that the contructor needs to be explicitly told, via IsRNA, what the backbone is because files do not store this information. Note also that save files explicitly store the thermodynamic parameters, therefore changing the backbone type as compaared to the original calculation will not change structure predictions.

**Parameters:**
:   |  |  |  |
    | --- | --- | --- |
    |  | *filename1* | is a null terminated c string and refers to sequence 1. |
    |  | *filename2* | is a null terminated c string and refers to sequence 2. |
    |  | *type1* | is an integer that indicates the file type for sequence 1. |
    |  | *type2* | is an integer that indicates the file type for sequence 2. |
    |  | *IsRNA* | is a bool that indicates whether these sequences are RNA or DNA. true=RNA. false=DNA. Default is true. Only one backbone is allowed for both sequences. |

|  |  |  |  |  |
| --- | --- | --- | --- | --- |
| TwoRNA::TwoRNA | ( |  | ) |  |

Constructor Default constructor that requires no parameters.

Constructor Default constructor that requires no parameters.

|  |  |  |  |  |
| --- | --- | --- | --- | --- |
| TwoRNA::~TwoRNA | ( |  | ) |  |

Destructor.

The destructor cleans up all memory allocation.

---

## Member Function Documentation

|  |  |  |  |  |
| --- | --- | --- | --- | --- |
| int TwoRNA::GetErrorCode | ( |  | ) |  |

Return an error code, where a return of zero is no error.

This function returns and error flag that is generated during construction by RNA(const char &filename, const int type, const bool IsRNA=true) or from CalculateFreeEnergy(). An error of zero is always no error. Other codes are errors and a c-string can be fetched for the error with GetErrorMessage().

**Returns:**
:   An integer that provides the error code.

|  |  |  |  |  |  |
| --- | --- | --- | --- | --- | --- |
| char \* TwoRNA::GetErrorMessage | ( | const int | *error* | ) |  |

Return error messages based on code from GetErrorCode and other error codes.

0 = no error 1000 = Error associated with sequence 1 or with a procedure, function will get message from sequence 1 (the inherited RNA class). 2000 = Error associated with sequence 2, function will get message from sequence 2 (the RNA2 class). 3000 = Errors with each sequence, function will get messages from each.

**Parameters:**
:   |  |  |  |
    | --- | --- | --- |
    |  | *error* | is the integer error code provided by GetErrorCode(). |

**Returns:**
:   A pointer to a c string that provides an error message or from other functions that return integer error codes.

Reimplemented in Dynalign\_object.

|  |  |  |  |  |  |
| --- | --- | --- | --- | --- | --- |
| std::string TwoRNA::GetErrorMessageString | ( | const int | *error* | ) |  |

Return error messages based on code from GetErrorCode and other error codes.

Although RNA generally uses c strings, this member function returns a string that is suitable for interfacing with JAVA, etc. See the error list in the GetErrorMessage() entry.

**Parameters:**
:   |  |  |  |
    | --- | --- | --- |
    |  | *error* | is the integer error code provided by GetErrorCode() or from other functions that return integer error codes. |

**Returns:**
:   A string that provides an error message.

|  |  |  |  |  |
| --- | --- | --- | --- | --- |
| RNA \* TwoRNA::GetRNA1 | ( |  | ) |  |

return A pointer to the underlying structure class for sequence 1.

Access the underlying RNA class. This is provided for use with two sequence methods. Generally, there is no need for end users to use this function.

**Returns:**
:   A pointer to the underlying RNA class for sequence 1.

|  |  |  |  |  |
| --- | --- | --- | --- | --- |
| RNA \* TwoRNA::GetRNA2 | ( |  | ) |  |

return A pointer to the underlying structure class for sequence 2.

Access the underlying RNA class. This is provided for use with two sequence methods. Generally, there is no need for end users to use this function.

**Returns:**
:   A pointer to the underlying RNA class for sequence 2.

|  |  |  |  |  |
| --- | --- | --- | --- | --- |
| double TwoRNA::GetTemperature | ( |  | ) |  |

Get the current folding temperature in K.

**Returns:**
:   A double that indicates the folding temperature in K.

|  |  |  |  |  |  |
| --- | --- | --- | --- | --- | --- |
| int TwoRNA::SetTemperature | ( | double | *temperature* | ) |  |

Set the temperature at which the calculation will be performed in K.

This function allows the user to specify folding temperatures other than 310.15 K (37 degrees C). This changes folding free energy changes that would be returned for existing structures and would alter the set of structures predicted. When this function is called, the thermodynamic parameter files are immediately read from disk. These include both enthalpy parameters (.dh files) and free energy changes at 310.15 (.dat files). The files must either be at a location indicated by the $DATAPATH environment variable or in pwd. Changing the temperature only alters subsequent calculations. For example, if a structure prediction method has been called, the set of predicted structures are not changed at the time of a call to SetTemperature. Likewise, SetTemperature must be called before calling a structure prediction method if a temperature other than the 310.15 K default is desired. The function returns an error code where 0 is no error and non-zero errors can be parsed by by GetErrorMessage() or GetErrorMessageString() in an inheriting class.

---

## Member Data Documentation

char TwoRNA::compoundmessage[COMPOUNDMESSAGELENGTH]

Provide facility for storing and accessing error messages.

int TwoRNA::ErrorCodeTwo `[protected]`

RNA\* TwoRNA::rna1 `[private]`

RNA \* TwoRNA::rna2 `[private]`

---

The documentation for this class was generated from the following files:

- F:/RNAstructure-co-from-repository/RNAstructure/RNA\_class/TwoRNA.h- F:/RNAstructure-co-from-repository/RNAstructure/RNA\_class/TwoRNA.cpp

---

Generated on Tue Jul 30 10:52:29 2013 for RNAstructure Classes by 
 1.5.7.1
